# The Effects of Environmental Factors on Persons Living with HIV/AIDS

**DOI:** 10.3390/ijerph6072041

**Published:** 2009-07-23

**Authors:** Lucersia Nichols, Paul B. Tchounwou, Leandro Mena, Daniel Sarpong

**Affiliations:** 1 NIH-RCMI Center for Environmental Health, Jackson State University, Jackson, MS, USA; E-Mail: paul.b.tchounwou@jsums.edu; 2 University of Mississippi Medical Center, Jackson, MS, USA; E-Mail: Lmena@medicine.umsmed.edu; 3 Jackson Heart Study, Jackson State University, Jackson, MS, USA; E-Mail: daniel.f.sarpong@jsums.edu

**Keywords:** Human Immunodeficiency Virus (HIV), Acquired Immunodeficiency Syndrome (AIDS), Sexually Transmitted Disease (STD) patients, environmental barriers, quality of life

## Abstract

In recent years, environmental awareness has received a great deal of public attention. However, little emphasis has been put on the influence of environmental factors (weather, personal attitudes, policies, physical structures, transportation, etc.) on the quality of life of persons infected with HIV/AIDS. The goal of this study was to assess the effect of selected environmental factors on the quality of life of persons affected by HIV/AIDS. To achieve this goal, the Craig Hospital Inventory of Environmental Factors (CHIEF) subscales including *Policies, Physical Structure, Work/School, Attitudes/Support, and Service/Assistance* were evaluated in patients selected from a STD/HIV clinic in Jackson, MS. They were chosen based on previously diagnosed HIV/AIDS status and age (16–95). Written consents, demographics sheets and self-administered questionnaires were obtained. Data were analyzed using Excel and SPSS software. Interviews started in July 2007 and ended in August, 2007. One hundred and thirteen patients responded. Participants were 72.6% (82) male, 26.5% (30) female and 0.9% (1) transgender. The median age of participants was 38.8 (18–63). Over 50% (65) had some college or higher education, and 35.4% reported annual incomes less than $10,000. Multivariate analysis showed marginal significance between disease diagnosis and gender (*p <* 0.10), and statistical significance between disease diagnosis and income (*p =* 0.03). Also, age (*p =* 0.01) and education (*p =* 0.03) were significant predictors in one of the subscales. The CHIEF subscales that showed the greatest significance among AIDS respondents were *Attitudes and Support*, and *Government Policies* with mean sensitivity scores of 1.39 and 1.42, respectively. The element with the least effect on AIDS patients was the *Work/School* subscale, with a mean score of 0.74. In general AIDS patients were disproportionately affected in all but one of the five subscales observed. Conversely those with HIV were more affected in the *Work/School* subscale with a mean score of 1.70. This proved to be the only subscale responsible for causing the greatest degree of perceived barriers for the HIV population. With a mean score of 0.75, *Physical/Structural* subscale showed the least negative impact on those infected HIV without AIDS. It is therefore recommended that the environmental barriers identified in this study be addressed in order to eliminate/minimize their negative effect and improve the quality of life of HIV/AIDS patients.

## 1. Introduction

When Acquired Immune Deficiency Syndrome (AIDS) was first recognized in 1981, patients with the disease were unlikely to live longer than one or two years. Since then, scientists have developed an effective arsenal of drugs that help manage the Human Immunodeficiency Virus (HIV), so that persons infected with the virus can live longer and healthier lives. Although there is currently no vaccine or cure for HIV or AIDS, the development of Highly Active Antiretroviral Treatment (HAART) as effective therapy for HIV infection and AIDS has substantially reduced the death rate from this disease. As the life expectancy of persons with AIDS has increased in countries where HAART is widely used, the number of persons living with AIDS has increased substantially [1]. Globally, there were an estimated 33 million [30–36 million] people living with HIV in 2007 [2]. An alarming fact was that in the United States alone the number of persons with AIDS increased from about 35,000 in 1988 to more than 220,000 in 1996, an increase of over 180,000 in less than 10 years [3]. Approximately 11 years later, by the end of 2007, the CDC estimated that the number of persons living with AIDS had reached 468,578, with 56,300 new HIV infections occurring annually [4,5]. According to data obtained from a 2005 Mississippi Living HIV Disease Report, as of December 31, 2005, Mississippi accounted for a total of 8,330 cases of HIV Disease. An estimated 3,347 (40.2%) were located in Hinds County. Also as of 2007, Mississippi accounted for 2% of the national total of HIV cases reported (4,953 of 337,590) [6,7].

While the federal government’s investment in treatment and research is helping people with HIV/AIDS live longer and more productive lives, HIV continues to spread at staggering rates. In a study named the HIV Aware/Not in Care Project several environmental/structural barriers to productive living were identified. The environmental barriers identified included; hassle of getting care, negative provider patient relationships, societal attitudes, and funding for care. Structural barriers to successful HIV treatment were transportation and poverty. The social barriers identified included; care-giving responsibilities (putting others first), fear of stigma and discrimination, and disclosure concerns [8,9]. This presents a clear and evident problem. Research needs to redirect some of its attention toward a more thorough investigation of environmental factors as barriers to productive living for those infected with HIV/AIDS.

Previous research based on the Craig Hospital Inventory of Environmental Factors has demonstrated that various environmental factors including transportation issues, weather, finances and societal attitudes serve as barriers to the productive living of persons with disabilities such as traumatic brain injury, spinal chord injury, amputations and others. However, very little research has been done on environmental barriers related to HIV/AIDS. In the present study, we hypothesize that specific environmental factors/conditions have a negative impact on the quality of life of persons suffering from HIV infection and/or AIDS. Hence, our main objective was to assess and identify environmental factors that are perceived by HIV/AIDS patients as impediments to productive living.

With the advent of HAART, HIV infected persons can focus not only on the treatment’s ability to extend their life span but also their quality of life. For the purpose of this article quality of life has been defined as the personal satisfaction (or dissatisfaction) with the cultural or intellectual conditions under which you live [10].

## 2. Materials and Methods

### 2.1. Questionnaire Design and Administration

Although several methods of conceptualizing environmental factors and their relationship to disability have been suggested, Fougeyrolles was the first to offer taxonomy or listing of environmental factors. He and the Canadian Society for the International Classification of Impairments, Disabilities and Handicaps (ICIDH) cataloged multiple elements of the environment that are viewed as important determinants of handicap or participation [11]. This strategy has been incorporated into the current classification scheme of the environment included in the beta draft of the ICIDH-2 [12]. This strategy has been identified as providing an exhaustive list of environmental elements that may influence the disablement process, but it does not prove to be useful as a conceptual framework for quantifying the environment in survey tools. Alternatively, Whiteneck *et al*. identified five characteristics of the environment that corresponded with or helped facilitate participation by persons with disabilities. The five characteristics of the environment purposed in the CHIEF manual were listed as: accessibility, accommodation, resource availability, social support and equality. Each is spelled out and defined in the manual as follows: Accessibility answers the question “Can you get where you want to go?” Accommodation addresses the question “Can you do what you want to do?” Resource availability addresses the question of, “Are your special needs met?” Social support addresses the question “Are you accepted and supported by those around you?” Finally, equality addresses the question “Are you treated equally with others?” These five characteristics have been found to be useful tools in evaluating environmental influences. Despite this they must be observed on an individual basis and applied to each individual’s own situation. What could potentially restrict one person may have the opposite effect and assist or not affect another. In each case, these five environmental characteristics can be assessed ranging from restrictive barriers to inclusive facilitators [13].

The Craig Hospital Inventory of Environmental Factors (CHIEF) model was chosen for this study largely based on the fact that it had been pre-validated and contained questions that are instrumental in acquiring the data necessary to evaluate the effect of environmental factors on persons living with HIV/AIDS. In addition to questions related to environmental barriers, care was taken to add questions to collect demographic information including age, sex, and race. Hence, the CHIEF protocol was administered to HIV/AIDS patients to collect relevant environmental information including barriers related to: (a) school and work barriers, (b) physical and structural settings, (c) attitudes and support, (d) government policy barriers, and (e) services and assistance. These items individually encompass all of the areas specifically identified as barriers and as such have been found to be contained within the framework of the CHIEF questionnaire. Therefore, the CHIEF model asks questions designed to track the frequency (how often are they encountered?) and magnitude (how severe are they?) of each potential environmental barrier. From the study participants responses, the frequency at which barriers are encountered are calculated based on a scale of 0–4 (0 = never, 1 = less than monthly, 2 = monthly, 3 = weekly, and 4 = daily), the magnitude of the problem related to a specific barrier is rated on a scale of 0–2 (0 = no problem if the barrier has never been encountered, 1 = a little problem, and 2 = a big problem), and the overall score representing the product of frequency times magnitude is presented on a scale of 0–8 indicating the overall impact of the barrier. Questions were prefaced by the words, ‘In the past 12 months how often has…, where respondents could shade in one of the five answers given. As it relates to work or school they could choose a sixth response of “Not Applicable”. Once the answer was chosen as to how often they were then guided to a second part of the same question stating, “When this problem occurs has it been a big problem or a little problem”. Respondents could then shade in one of the two (questionnaire attached). Detailed information on questionnaire administration and scoring is outlined in the CHIEF manual [www.craighospital.com].

### 2.2. Study Site

The Mississippi Department of Health (MDH) Crossroads Clinics is the state agency primarily responsible for providing HIV/AIDS screening, diagnosis and partner referral services in Mississippi. MDH is joined by the Division of Medicaid (DOM) and the University of Mississippi Medical Center (UMMC) as state-level agencies that provide HIV/AIDS services. Medicaid funds the majority of all HIV/AIDS services provided in Mississippi. The MDH partners with UMMC through its pediatric, adolescent, adult, and maternal HIV clinics. Together, these three agencies provide nearly all the care for medically indigent people living with HIV in Mississippi. The MDH Crossroads Clinics alongside its Field Services office was recruited as the research site primarily because of the afore mentioned statistics, being located in the center of the greater Jackson metropolitan area (Hinds County), and the fact that in the “2005 Mississippi Living with HIV Disease Report”, of the nine districts surveyed, District Five (Hinds County) had the highest rating of over 40% of the total cases of HIV [6].

Crossroads Clinics is also a site for The Early Intervention Program whose goals are to provide comprehensive evaluation to newly diagnosed HIV infected clients attending the clinic and to facilitate transfer of Crossroads clients newly diagnosed with HIV infection to a definitive HIV provider. During this process, clinic staff such as disease intervention specialists, case managers and clinicians do a complete evaluation of the patient to determine any and all potentially important client needs, from counseling of patient about HIV status, to completion of any needed laboratory tests, to referring clients to either Private Medical Doctor (PMD) of choice or to UMMC for continued HIV primary care. In addition to all of this, Crossroads Clinics was also the site for distribution of Ryan White Title II funded medications and enrollment in The Housing Opportunities for Persons with AIDS (HOPWA) program. Once the study site had been chosen the next step involved acquiring the required approvals necessary for conducting a research study. Prior to project initiation, the research proposal, IRB application, survey questionnaire, consent form and demographics sheet were submitted for review to the Institutional Review Boards (IRB) of the Mississippi State Department of Health (MSDH) and Jackson State University (JSU). Once both approvals had been obtained, the primary researcher, case manager and/or medication nurses were cleared to begin soliciting patients for the study. The process of selecting participants was left up to the case manager, the primary researcher or the medication nurse for the clinic. Due to the fact that the case manager and the medication nurse had previously been trained in Health Information Portability and Accountability Act (HIIPAA) rules and regulations and were state employees the right to view patient medical records required no additional provisions be taken.

### 2.3. Sampling Process

Based on multiple unsuccessful attempts to find research where the CHIEF was used in the field of HIV/AIDS it was determined that this study should be viewed as a pilot study with the aim of specifically applying the CHIEF survey tool to a new sample population, namely HIV/AIDS. Sampling was subsequently based on patient volume during the months of July and August 2007. According to data from Crossroads Clinic an average of 34 HIV/AIDS patients were seen weekly and for the allocated time frame between 100–120 patients could be surveyed. This 100–120 persons would be divided into two categories; 50–60 HIV positive persons (no AIDS) and 50–60 HIV positive persons (with AIDS). Inclusion criteria were: documented evidence of a previously diagnosed HIV infection from a medical provider and age of at least 16 but no older than 95 years [14]. Exclusion simply meant there was no documented diagnosis of HIV infection or the age requirement was not met. For those with AIDS there also had to be documented evidence by a medical provider stating that this person had a confirmed diagnosis of AIDS. The exclusion criteria were also based on the lack of documented proof that the individual had been diagnosed with AIDS and/or not meeting the age requirement. In all cases CD4 count was examined and recorded (if available) to correlate with the HIV or AIDS diagnosis. A CD4 count of less than 200 at any given point during the disease stage would constitute a diagnosis of AIDS [15]. If at some point because of ARV’s the CD4 count became greater than 200, the diagnosis of AIDS remained the same. Convenient samples were taken of HIV/AIDS patients entering the Crossroads Clinics for services that included medication pick-up and dental visits. Participants were approached and taken to a private setting where the primary researcher, case manager or nurse would first acquire verbal consent. After that a written consent, previously signed by the researcher, would be signed by the patient. Once signed consent was obtained, the questionnaire was explained starting with the first set of questions giving a brief overview and then moving on to the body of the questionnaire. Once the questionnaire had been explained, the patients were shown the demographics sheet located at the back of the questionnaire and advised to complete this along with the questionnaire. Those patients who were capable of filling out the questionnaire were left to do so with periodic checks from the researcher. For those incapable of reading the questionnaire it was verbally administered by the primary researcher.

### 2.4. Data Collection

To be sure that no patient rights were violated according to HIPAA, all of the data collected from the patients had to be handled with utmost care. Once the consent form was signed by both parties it was placed in the patient’s medical record where it would remain for the 2-year required time frame and afterward shredded. Despite the fact that no identifying information was contained in the questionnaire, it was placed in a file designated either HIV or AIDS and then retained under lock and key in a protected area. For accuracy sake once the patient finished a questionnaire it was coded as follows: A-1 thru A-50, for the patients with AIDS and, H-1 thru H-50, for the patients with HIV no AIDS. This approach was used to insure accuracy when inputting data as well.

### 2.5. Data Analysis

A total of 113 questionnaires were completed during the months of July and August 2007. Each questionnaire took approximately 15 minutes to complete and out of 116 patients approached, three declined. Multivariate linear regression model assessed factors associated with the total CHIEF and six sub-scales as the response variable. Means and standard deviations were computed for continuous variables and percentages were computed for categorical variables. Frequency distributions i.e., percentages, were computed for each item of the CHIEF and were stratified by disease diagnosis status. Mean product scores (i.e., mean of the product of the frequency and magnitude) for each item of the CHIEF were computed for each disease diagnosis. Differences in the mean product scores were assessed using analysis of variance (ANOVA) tests. Microsoft Excel was used to input the data into a workable data base. A data dictionary was structured so that when the numbers were analyzed, they would correlate with the scoring system initially set up by the CHIEF. Once the data had been entered into the data base, it was analyzed using Statistical Package for the Social Sciences (SPSS) to calculate standard deviations and means for each CHIEF item, sub-scale and total score. ANOVA was also used for difference in mean product scores. To differentiate between both groups, HIV and AIDS, data were analyzed to show differences in reported frequency and magnitude of environmental barriers across the various sub-scales.

## 3. Results

### 3.1. Study Population

Interviews started in July, 2007 and ended in August, 2007. One hundred and thirteen patients responded. Participants were 72.6% (82) males, 26.5% (30) females and 0.9% (1) transgender. The median age of participants was 38.8 (18–63). Respondents were 84% (95) African Americans, single 62% (71) individuals. Over 50% (65) had some college or higher education, but 35.4% reported incomes less than $10K yearly. Multivariate analysis showed marginal significance between disease diagnosis and gender (*p <* 0.10), statistical significance between disease diagnosis (*p =* 0.02) and income (*p =* 0.03).

### 3.2. CHIEF Policies Subscale

**Figure 1.**
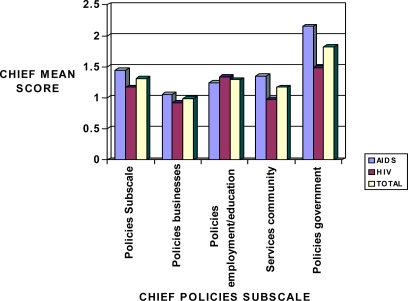
Sensitivity mean scores in HIV and AIDS patients in relation to the CHIEF Policies subscale.

Data obtained for the Polices subscale including business, employment/education, community and government policies are presented in Figure 1. The overall mean score for the combined HIV and AIDS was 1.42 indicating that Policies in general are perceived by HIV/AIDS patients as having an impact on their quality of life. Government policies had a highly significant impact (mean score of 2.10) compared to other types of policies (Figure 1).

### 3.3. CHIEF Physical/Structural Subscale

**Figure 2.**
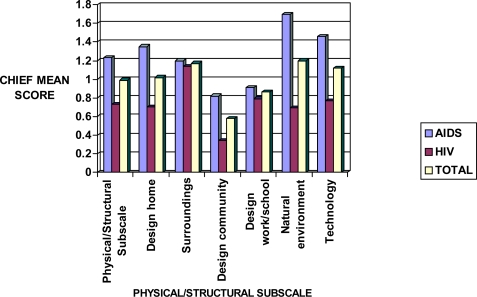
Sensitivity mean scores in HIV and AIDS patients in relation to the CHIEF Physical/Structural subscale.

Physical/structural subscale showed little impact on both HIV and AIDS patients, with an overall mean score of 0.98 (Figure 2). The categories of this subscale included design home, surroundings, design community, design work/school, natural environment, and technology. Significant variations were found in the level of perceived risks associated with these categories in both HIV and AIDS participants.

### 3.4. CHIEF Work/School Subscale

**Figure 3.**
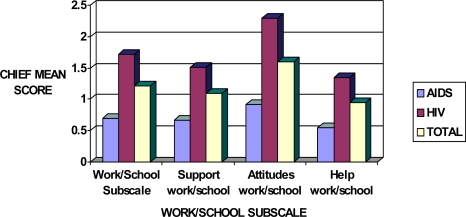
Sensitivity mean scores in HIV and AIDS patients in relation to the CHIEF Work/School subscale.

Work/school subscale was shown to have the least effect on AIDS patients compared to those infected with the HIV only. The mean scores in all categories including support, attitudes and held were significantly higher in HIV than in AIDS patients (Figure 3). The overall sensitivity mean scores were 1.68 and 0.60 in HIV-infected persons and AIDS patients, respectively.

### 3.5. CHIEF Attitudes and Support Subscale

**Figure 4.**
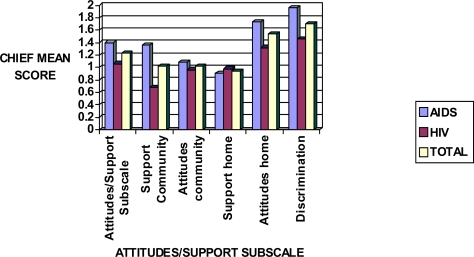
Sensitivity mean scores in HIV and AIDS patients in relation to the CHIEF Attitudes/Support subscale.

Attitudes/Support subscale showed the greatest significance among AIDS respondents; with a mean score of 1.38. Discrimination proved to have the greatest effect in this subscale but those with AIDS also perceived barriers with attitudes at home (Figure 4).

### 3.6. CHIEF Services/Assistance Subscale

**Figure 5.**
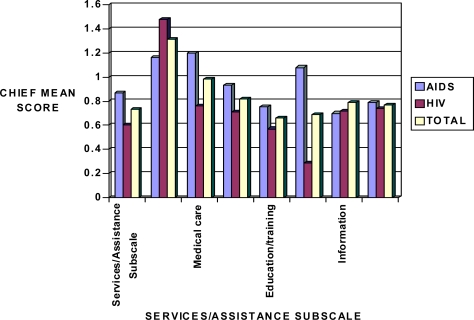
Sensitivity mean scores in HIV and AIDS patients in relation to the CHIEF Services/Assistance subscale.

Overall, persons with AIDS disease indicated the most perceived barriers were perceived in medical care, transportation, and help in the community categories. Transportation posed the greatest problem for both HIV and AIDS groups, but proved most problematic for those with HIV (Figure 5).

### 3.7. CHIEF Overall/Total Subscale

**Figure 6.**
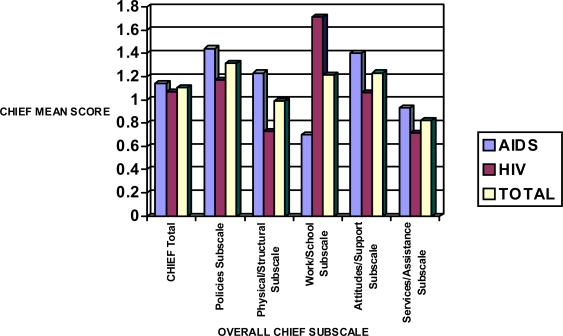
Sensitivity mean scores in HIV and AIDS patients in relation to CHIEF Subscales.

Figure 6 presents the integrated data for all the five CHIEF subscales evaluated. Data presented in this figure indicate that except for the Work/School subscale, AIDS patients were disproportionately affected, and hence perceived higher environmental barriers to productive life, with regards to policies, physical/structural, attitudes/support, and service/assistance. The Work/School subscale seemed to have the greatest level of perceived barriers for those infected with HIV. Age (*p =* 0.01) and education (*p =* 0.03) were significant predictors of perceived environmental barriers in this subscale.

## 4. Discussion

The goal of this study was to utilize a survey-based research design and quantitative data analysis for the purpose of eliciting information relating to the effects of environmental factors on persons living with HIV/AIDS, and more specifically those HIV/AIDS patients attending the Crossroads Clinics for services. The study aimed at evaluating environmental factors that were self reported as barriers to productive living. The direct relationship between these factors and HIV/AIDS has been subject to previous research. The present investigation can now serve as forerunner in the development of more advanced studies dedicated to understanding how the environment may negatively impact the quality of life of persons living with HIV/AIDS.

The study objectives were to:

1. Identify the relationship between environmental factors and those infected with HIV/AIDS
2. Determine which environmental factors serve as barriers to productive living for those infected with HIV.
3. Determine if there exists a causal relationship between observed environmental barriers and specific descriptive characteristics of HIV/AIDS patients.

Findings from this study indicate that specific environmental conditions and barriers related to school and work, physical and structural settings, attitudes and support, government policy, and public services and assistance are perceived by HIV/AIDS patients as having a negative impact in their quality of life. In a similar study conducted by Craig researchers on 330 Colorado residents that included 55% of persons with disabilities and 44% without disabilities, 1,085 specific examples of environmental barriers were reported. For both people with disabilities and those without disabilities, the number one barrier was weather – both hot and cold temperatures and snow, sleet, and ice. The second most common barrier reported by both groups was lack of family support [16]. Another community-based study conducted in a disabled population pointed out that amputees were more likely to perceive barriers in all areas except work/school. This study also reported that perceived environmental barriers were highly prevalent among persons with limb loss compared to non-disabled Americans [17].

Another CHIEF-based study has been performed on persons with traumatic brain injuries (TBI) to determine the types of environmental barriers reported and to identify the relations between environmental barriers and such components of societal participation as employment, community mobility, social integration, and life satisfaction. The barriers identified to have the greatest impact included transportation, environmental surroundings, government policies, attitudes and the natural environment. It was noted that respondents who were married, older, and unemployed, or not in school reported the most barriers overall. It was concluded that although environmental barriers affect TBI survivors and play a role in their outcomes, their interplay with others, perhaps as yet unidentified, factors require continued research. It was also agreed that the CHIEF may be a valuable tool for understanding the environment’s role in the lives of people with TBI and identifying the general environmental domains where interventions are needed to reduce their negative impact [18].

Although the CHIEF was designed to measure factors that keep people from getting things done, it is well known that the type and severity of a disability can affect what an individual is able to accomplish. Also, both intrinsic (health status, weight, education, attitudes and motivation) and extrinsic (physical surroundings and accessibility, attitudes and support of others, available resources, rules, regulations and government policies) factors may have a significant impact on the lives of people with disabilities. In conclusion, the information collected in our CHIEF-based surveys provided a basis to understand how HIV/AIDS patients are affected by their environment, and how the specific characteristics of this environment impact their quality of life. Ultimately, this information is instrumental in helping policy makers implement the changes needed to minimize the public health impact.

The findings outlined by the study done on those with traumatic brain injuries showed similar findings to this study, although done on persons with HIV/AIDS. In this study, it was also found that transportation, the surroundings, government policies, attitudes and the natural environment were considered by this group of people, particularly those with AIDS, to be the items that showed the greatest perceived barriers.

Whiteneck’s evaluation of the CHIEF as a valuable tool when used to evaluate the effects of specified environmental factors is substantiated by this study. Despite this truth further studies designed to address specific factors of the environment need to be done where a larger sample size is chosen and follow-up questionnaires can be administered over a longer period of time. These findings prove instrumental in showing that the environment plays a significant role in the lives of those infected with HIV/AIDS.

### 4.1. Limitations

Almost 90% of the participants were African Americans which reduces the possibility of generalizability. Future studies should consider a more representative racially diverse population conducted in diverse settings. Further, the majority of the sample was male, thereby not accounting for gender differences. Also, Participants were recruited from one community center. Findings may be varied for persons living with HIV and/or AIDS who have private physicians or other health care providers.

At certain stages, AIDS is known to be a very debilitating disease; therefore those with AIDS have to sometimes rely on others to carry out simple daily responsibilities. At one point in the study it was noted that the number of new AIDS participants started to decline, indicating that the pool of AIDS candidates had been virtually exhausted. Knowing this, future studies should involve multiple study sites, in an attempt to acquire a larger number of AIDS participants. Further, health takers who provide services to persons living with HIV and/or AIDS should be provided education on the types of environmental factors identified as barriers to productive living.

Based on self-reported data, it was also found that questionnaire understanding plays an important role. Some participants did not fully understand the instructions, as indicated by missing data. Most incidences of missing data came from the design and formulation of questions. Each question had two parts and respondents with the missing data failed to fill out one of the two parts thus implying that more time should be allocated to explaining the questionnaire before hand and evaluating it afterwards.

Income level was also a limiting factor because the Crossroads Clinics HIV/AIDS programs are specifically designed for those individuals who fall within a specific income range. Those with higher incomes were underrepresented creating a bias towards the lower income population. Moreover, the sample size included in the multivariate linear regression analysis may have been too small to detect or ascertain important factors that determine or predict the total CHIEF and respective sub-scales.
